# Prehospital Detection of Large Vessel Occlusion and Intracerebral Hemorrhage Using a Dual-Biomarker Point-of-Care Test

**DOI:** 10.1161/SVIN.125.002170

**Published:** 2026-03-02

**Authors:** Arnab Ghosh, Noah L.A. Nawabi, Diana Alcedo, Rodolfo E. Alcedo Guardia, Juan Vicenty-Padilla, Saef Izzy, Nirav J. Patel, Rose Du, Adam A. Dmytriw, Alfred P. See, Mohammed Ali Aziz-Sultan, Toby I. Gropen, David Liebeskind, Erickson F. Torio, Anil Can, Lennard Spanehl, Edoardo Gaude, Joshua D. Bernstock

**Affiliations:** 1 Medical Sciences Division, University of Oxford, United Kingdom (A.G.).; 2 Computational Neuroscience Outcomes Center, Department of Neurosurgery, Brigham and Women’s Hospital, Harvard Medical School, Boston, MA (N.L.A.N.).; 3 Neurointerventional and Neuroanalytics Collaboration, School of Medicine, Toronto Metropolitan University, ON, Canada (A.A.D.).; 4 College of Medicine, Medical University of South Carolina, Charleston (N.L.A.N.).; 5 Ponce Health Sciences University, Puerto Rico (D.A.).; 6 Neurosurgery Section, School of Medicine, University of Puerto Rico, Medical Sciences Campus, San Juan (R.E.A.G., J.V.-P.).; 7 Department of Neurology (S.I.), Brigham and Women’s Hospital, Boston, MA.; 8 Department of Neurosurgery (N.J.P., R.D., M.A.A.-S., E.F.T., A.C., L.S., J.D.B.), Brigham and Women’s Hospital, Boston, MA.; 9 Department of Neurosurgery, Boston Children’s Hospital, MA (A.P.S.).; 10 Department of Neurology, The University of Alabama at Birmingham Heersink School of Medicine (T.I.G.).; 11 Comprehensive Stroke Center and Department of Neurology, University of California, Los Angeles (D.L.).; 12 Pockit Diagnostics Ltd, University of Cambridge, CRUK Cambridge Institute, United Kingdom (E.G.).

**Keywords:** biomarkers, cerebral hemorrhage, glial fibrillary acidic protein, predictive value of tests, triage

## Abstract

**BACKGROUND:**

Timely identification of stroke subtype is critical for triage and treatment. Existing prehospital stroke scales have limited diagnostic accuracy, especially in differentiating large vessel occlusion (LVO) from intracerebral hemorrhage (ICH). The LVOne point-of-care test measures d-dimer and GFAP (glial fibrillary acidic protein), biomarkers associated with thrombotic and hemorrhagic stroke, enabling biologically informed triage.

**METHODS:**

We validated a new version of the LVOne assay using plasma samples from 210 suspected stroke patients presenting within 6 hours of symptom onset, enrolled in the TIME (Testing of Identification Markers for Stroke) prospective observational cohort. This article reports a retrospective (secondary) laboratory analysis of these prospectively collected samples and clinical data. Samples were collected before neuroimaging or thrombolysis. LVOne version 1 combined d-dimer positivity, GFAP negativity, and Field Assessment Stroke Triage for Emergency Destination scoring for LVO detection only; version 2 uses improved GFAP measurement and a clinical decision rule enabling simultaneous diagnosis of LVO and ICH. Diagnostic performance (sensitivity, specificity, positive predictive value, negative predictive value) was calculated for each version. Receiver operating characteristic analyses and biomarker correlations with Field Assessment Stroke Triage for Emergency Destination were also performed.

**RESULTS:**

Both LVOne versions achieved high accuracy for LVO detection (sensitivity, 0.75; specificity, 0.92; negative predictive value, 0.97). Version 2 improved ICH specificity (0.99 versus 0.90) and positive predictive value (0.83 versus 0.23) versus version 1. Biomarker levels correlated with Field Assessment Stroke Triage for Emergency Destination scores but with limited explanatory power (GFAP *R*^2^=0.077; d-dimer *R*^2^=0.047), suggesting added information beyond clinical severity scales. In a specimen-matrix comparison (n=25), qualitative GFAP/d-dimer classification was concordant across plasma, venous whole blood, and capillary whole blood.

**CONCLUSIONS:**

The updated LVOne assay enables simultaneous biomarker-based identification of both LVO and ICH in suspected stroke patients. Its rapid, low-complexity format supports prehospital use and may improve triage, reduce time to treatment, and optimize outcomes. Prospective field validation is warranted.

**REGISTRATION:**

URL: https://www.clinicaltrials.gov; Unique identifier: NCT04292600.

## GRAPHIC ABSTRACT

**Figure:**
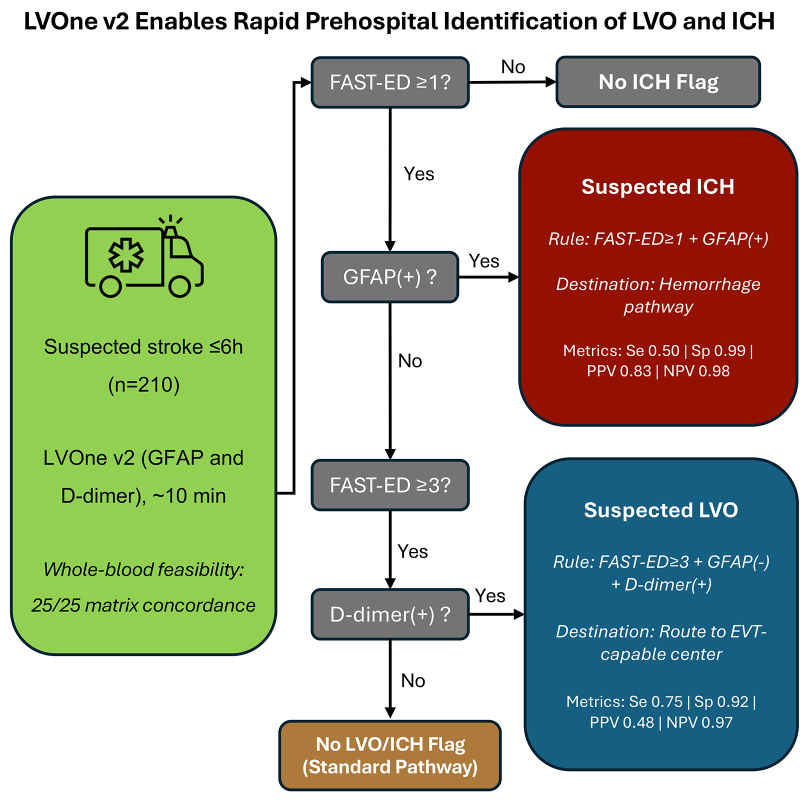

**CLINICAL PERSPECTIVE**

**What Is New?**

- LVOne version 2 integrates d-dimer and GFAP (glial fibrillary acidic protein) with clinical scales in a unified decision rule that enables simultaneous identification of large vessel occlusion and intracerebral hemorrhage from a single point-of-care test.
- Compared with the first-generation assay, version 2 demonstrates a marked improvement in intracerebral hemorrhage rule-in performance, with higher specificity and positive predictive value while preserving robust detection of large vessel occlusion.

**What Are the Clinical Implications?**

- A rapid dual-subtype point-of-care adjunct to clinical stroke scales is positioned to support prehospital triage and clinical decisions, potentially reducing transfer delays and optimizing clinical outcomes for patients with large vessel occlusion and/or intracerebral hemorrhage.

Stroke arises from a sudden disruption of cerebral blood flow, either due to vascular occlusion (ischemic stroke) or rupture (hemorrhagic stroke). Globally, there were an estimated 12 million incident strokes and 7 million related deaths in 2019 alone.^1^ The global value of lost welfare reached $2.06 trillion in that year, with ≈90% attributed to ischemic stroke and intracerebral hemorrhage (ICH).^2^ These figures reflect not only direct healthcare costs but also indirect and intangible losses from disability, informal caregiving, and reduced productivity. In the United States, the stroke-related value of lost welfare was estimated at $214.61 billion.^2^

Given these consequences, rapid identification and differentiation of stroke subtypes is essential for timely and effective treatment. Early recognition is particularly important because outcomes in large vessel occlusion (LVO) improve with faster delivery of endovascular thrombectomy (EVT), where each minute of delay reduces the chance of functional independence.^3^ Similarly, in ICH, early blood pressure reduction has been shown to improve outcomes in the hyperacute phase.^4^ Prehospital triage currently relies on clinical stroke scales such as Field Assessment Stroke Triage for Emergency Destination (FAST-ED), Rapid Arterial Occlusion Evaluation, or other clinical symptom scales, which assess deficits like facial droop, arm weakness, and speech disturbance to detect LVO. Their diagnostic performance remains suboptimal in real-world emergency medical services settings.^5^ For example, Rapid Arterial Occlusion Evaluation demonstrates a sensitivity of ≈60% and specificity of ≈81% for LVO in the field, while FAST-ED achieves somewhat higher sensitivity and specificity but lacks objectivity.^6^ Beyond limited accuracy for LVO detection, clinical symptom scales fail to differentiate between ischemic LVO and ICH, both requiring fundamentally different early interventions. To address this, biomarker-based point-of-care tests such as the LVOne device have been developed. LVOne measures GFAP (glial fibrillary acidic protein) and d-dimer—biomarkers previously validated for their diagnostic utility in stroke subtype classification.^7^ In LVO ischemic stroke, intravascular thrombus formation activates the coagulation cascade, followed by fibrinolysis, which releases d-dimer, a fibrin degradation product.^8^ GFAP, by contrast, is a structural protein released in response to astrocyte damage and is elevated in ICH and traumatic brain injury.^9^

LVOne is a lateral flow assay that provides results within 10 minutes from a capillary blood sample, making it suitable for prehospital deployment. When combined with FAST-ED, it has demonstrated 75% sensitivity and 92% specificity for LVO identification and helps exclude hemorrhagic stroke.^10^ However, earlier versions of the test were optimized primarily for LVO detection and lacked reliable thresholds for ICH classification. While neuroimaging remains the diagnostic gold standard, it is only available after hospital arrival. Thus, there is a critical need for accurate, rapid, and portable diagnostic tools to support prehospital stroke triage. A revised version of the LVOne assay has, therefore, been developed to address this gap, enabling simultaneous detection of both LVO and ICH.

This study aims to validate the diagnostic performance of the optimized LVOne assay using retrospectively collected samples from the TIME study (Testing of Identification Markers for Stroke), and to compare its classification accuracy across stroke subtypes.^7^ Early and accurate identification of LVO and ICH can enable faster, subtype-specific triage and treatment(s), including EVT for LVO or blood pressure control for ICH, ultimately improving outcomes and reducing morbidity/mortality.

## Methods

### Transparency and Openness Promotion Guidelines

In accordance with the American Heart Association Journals’ implementation of the Transparency and Openness Promotion Guidelines, the data and analytic code supporting the findings of this study are available from the corresponding author on reasonable request.

### Data Availability

Data are available upon reasonable request from the corresponding authors.

### Reporting Guideline

This study is reported in accordance with the STROBE Guidelines (Strengthening the Reporting of Observational Studies in Epidemiology; Supplemental Material).

### Study Design and Participants

**Figure 1.**
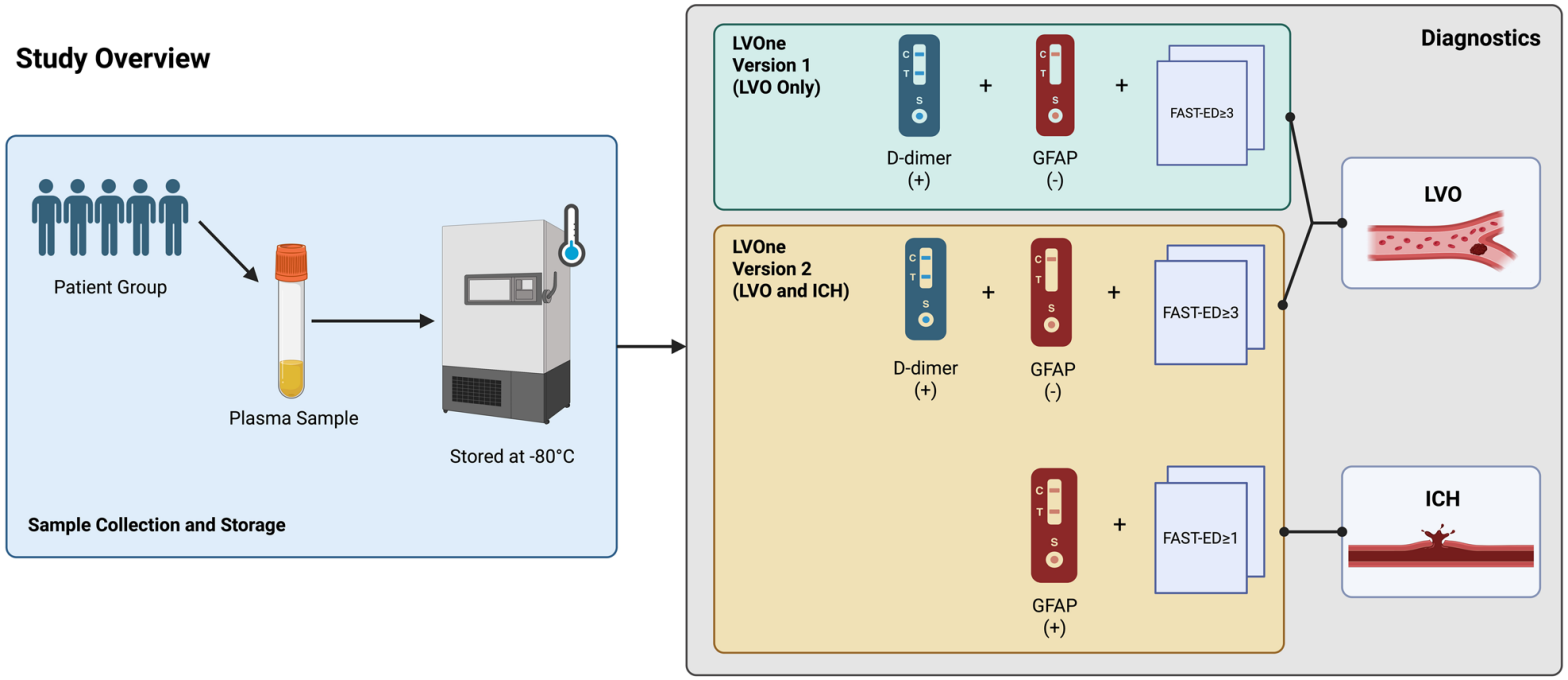
**Study overview and diagnostic workflow.** The figure illustrates a simplified testing workflow intended for prehospital or early in-hospital triage, designed to require minimal equipment and deliver results within ≈10–15 minutes in its intended point-of-care implementation (ie, from capillary whole blood). In this study, however, plasma samples were collected at initial evaluation, stored at −80 °C, and later analyzed retrospectively. Two diagnostic approaches were applied to the stored samples. LVOne version 1, as evaluated in Gaude et al,^10^ combined d-dimer and GFAP (glial fibrillary acidic protein) lateral flow assays with the Field Assessment Stroke Triage for Emergency Destination (FAST-ED) scale to identify large vessel occlusion (LVO), while using a negative GFAP result to exclude intracerebral hemorrhage (ICH). LVOne version 2 (current study) uses improved lateral flow technology for GFAP measurement and a revised diagnostic algorithm that enables separate rule-in of LVO and ICH and differentiation between these stroke subtypes. The workflow highlights integration of biomarker testing with clinical scales for early stroke subtype classification.

This study replicated the methodology of Gaude et al,^10^ with additional modifications (Figure 1). Plasma samples were obtained from the TIME study, which prospectively enrolled stroke alert patients presenting to Brandon Regional Hospital (Florida) within 18 hours of symptom onset, before administration of thrombolytic therapy or neuroimaging; full eligibility and exclusion criteria have been described previously.^7^. Patients were enrolled between May 2021 and August 2022. Inclusion criteria were (1) referred for acute suspected stroke and (2) time from known symptom onset within the last 18 hours. Exclusion criteria were (1) thrombolytic therapy received before blood collection, (2) symptoms known to have begun more than 18 hours ago, and (3) patient involved in a clinical trial for an investigational medicinal product. There was no longitudinal patient follow-up. In this study, a subset of the TIME cohort was used, that is, patients presenting within 6 hours from known symptom onset, as this is considered the time window where the test could have the most beneficial impact for acute stroke presentations. Missing data were minimal (sex: 1/210; arrival mode: 5/210). No imputation was performed; analyses were conducted using available-case data, with denominators varying by variable. Because the intended use of the test is to rule in patients with LVO or ICH, the study was powered on diagnostic specificity. With an expected prevalence of LVO or ICH of 10%, a minimum specificity of 80%, a 2-tailed type I error of 5%, and 90% power, a sample size of 138 suspected stroke patients would yield a specificity of 90% (95% CI, 80%–100%) for LVO or ICH. Venous whole blood was centrifuged at 4 °C, and plasma was stored at −80 °C until analysis. Laboratory analysis was performed in May 2025.

### Index Test (LVOne) and Biomarker Interpretation

For the present study, plasma samples were thawed and analyzed using the first- and second-generation LVOne tests according to manufacturer instructions. Briefly, 10 µL of plasma was applied to the test port, and the manufacturer’s instructions for LVOne (Catalogue number: PDL-002) were followed. GFAP was recorded as positive/negative based on the presence of a test line; d-dimer was scored visually using a colorimetric reference card by 2 blinded operators, with discordant results resolved by consensus. A d-dimer intensity score ≥2 was considered positive; any visible GFAP test line was considered positive.

### Prehospital Workflow and Specimen-Matrix Comparison

Although the primary validation in this manuscript was performed using archived venous plasma from the TIME biorepository, LVOne version 2 (v2) is intended for point-of-care use with finger-prick capillary whole blood in emergency settings, without the need for centrifugation or plasma separation by emergency medical services providers. To directly assess specimen-matrix compatibility, we performed an additional specimen-type comparison using venous plasma, venous whole blood, and finger-prick capillary whole blood (n=25 sample sets). Each specimen type was analyzed blank or after spiking with ×10 solutions containing known concentrations of d-dimer and GFAP, so that the resulting spiked sample would contain d-dimer or GFAP concentrations ≥600 ng/mL or 213 pg/mL, respectively, which are above the manufacturer’s positivity thresholds for the assay. LVOne v2 testing and interpretation were performed as above (GFAP: test line present/absent; d-dimer: visual score using the manufacturer's reference card and the prespecified positivity threshold).

### Clinical Decision Rule and Reference Standard

**Figure 2.**
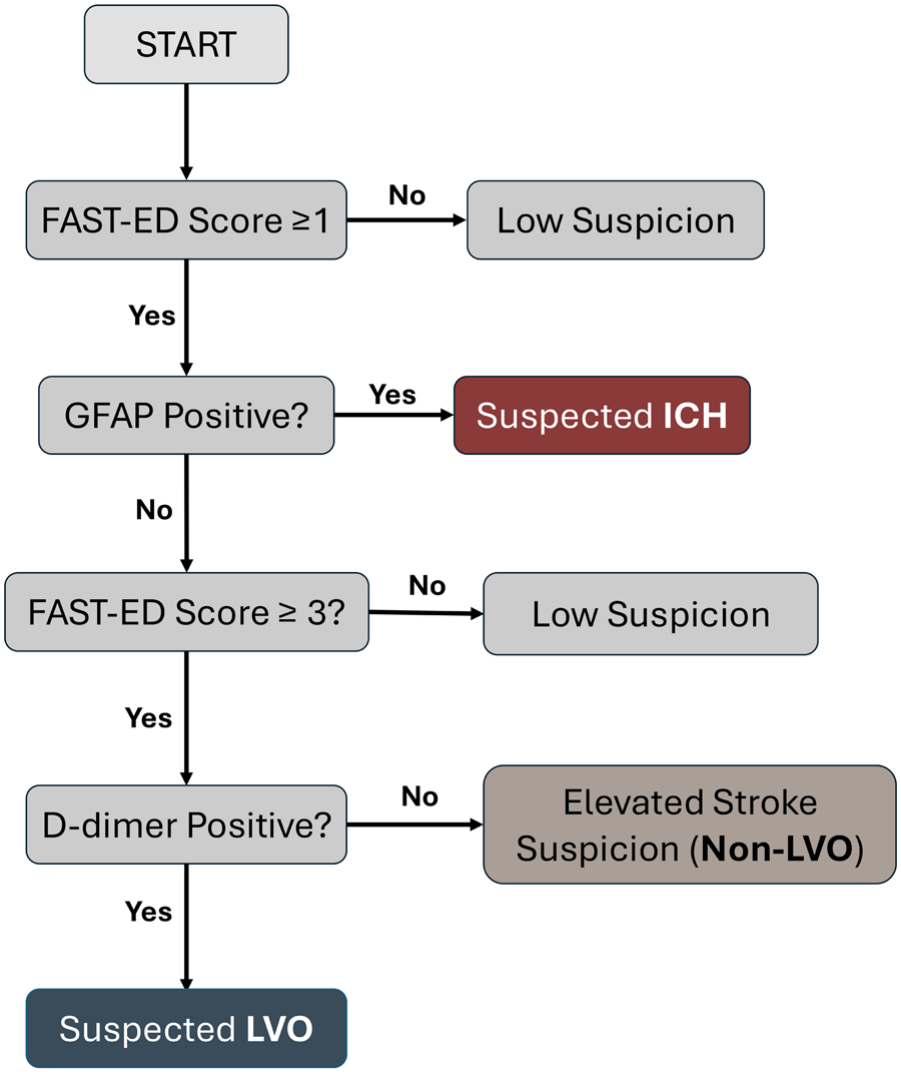
**LVOne decision algorithm for prehospital detection of large vessel occlusion (LVO) and intracerebral hemorrhage (ICH).** Flowchart illustrating the clinical decision pathway combining Fast Assessment Stroke Triage for Emergency Destination (FAST-ED) score, GFAP (glial fibrillary acidic protein), and d-dimer results. Patients with FAST-ED <1 are classified as low suspicion. Patients with FAST-ED≥1 and GFAP positivity are classified as suspected ICH. In GFAP-negative patients, d-dimer positivity combined with FAST-ED ≥3 indicates suspected LVO, while d-dimer positivity with FAST-ED<3 is classified as low suspicion. In GFAP-negative and d-dimer–negative patients, FAST-ED≥3 suggests elevated stroke suspicion (non-LVO), and FAST-ED <3 remains classified as low suspicion (69% positive predictive value for all-stroke (38/55 [95% CI, 56–80]), compared with 16% among FAST-ED <3 (25/155).

The clinical decision rule encompassed: (1) FAST-ED≥3+ d-dimer-positive+GFAP-negative, suggesting a suspected LVO, and (2) FAST-ED≥1+GFAP-positive, indicative of a suspected ICH (Figure 2). Final diagnostic category was assigned using neuroimaging and clinical records as per the TIME protocol.^7^ LVO was defined by computed tomography and angiography or magnetic resonance angiography confirmation of a large vessel arterial occlusion on a neuroradiology report (eg, ICA, vertebral/basilar, proximal MCA). ICH was defined by acute ICH on computed tomography or magnetic resonance imaging. Non-LVO ischemic stroke required exclusion of LVO on computed tomography and angiography/magnetic resonance angiography with supportive ischemic findings on magnetic resonance imaging, where available; transient ischemic attack/mimic diagnoses were assigned by expert clinical assessment with no pertinent imaging findings. Ethical approval was obtained (Western Institutional Review Board, 20193270).

### Statistical Analysis

Demographics were summarized using medians (interquartile range) for continuous variables and counts (percentage) for categorical variables. Global group differences were assessed using the Kruskal-Wallis test for continuous variables and χ^2^ or Fisher exact test for categorical variables. Where the global test was significant, group-vs-rest comparisons were performed (Mann-Whitney *U* test for continuous and Fisher exact test for categorical variables) with false discovery rate correction using the Benjamini-Hochberg procedure (*α*=0.05). Diagnostic performance (sensitivity, specificity, positive predictive value [PPV], negative predictive value [NPV]) was calculated with 95% CIs (Collett exact method). As a sensitivity analysis, we varied the LVOne v2 d-dimer visual intensity cutoff used to define a positive result (≥1, ≥2 [benchmark], ≥3), reapplied the triage rule (FAST-ED ≥3, d-dimer-positive, GFAP-negative), and recalculated performance metrics for LVO in the ≤6-hour cohort. Sensitivity analyses for LVOne version 1 (v1) d-dimer thresholds (≥3, ≥4, ≥5) were taken from the previously published Gaude et al,^10^ cohort and are presented for comparison only. We additionally fitted a univariable logistic regression model with ICH as the outcome and log10-transformed GFAP concentration (log10[GFAP+1]) as the predictor and plotted the predicted probabilities of ICH. Analyses were performed in R version 4.4.1.

### Modeled Triage Impact and Time-Outcome Translation

To contextualize operational impact, a secondary modeling analysis estimated expected triage outcomes per 1000 suspected stroke evaluations using (1) observed subtype prevalence in the TIME subset (LVO 20/210; ICH 10/210) and (2) diagnostic performance of LVOne v1/v2 from Table 2. For each strategy and target condition, expected counts were calculated as follows:

- True positive (TP)=1000×prevalence×sensitivity.
- False negative (FN)=1000×prevalence×(1−sensitivity).
- False positive (FP)=1000×(1−prevalence)×(1−specificity).
- True negative (TN)=1000−TP−FN−FP.

A scale-only current-standard comparator was included using published FAST-ED≥4 performance for LVO detection (sensitivity, 0.60; specificity, 0.89).^11^

To estimate the potential clinical impact of earlier EVT-capable routing, a time-saved scenario was applied using the Systematic Evaluation of Patients Treated With Neurothrombectomy Devices for Acute Ischemic Stroke (STRATIS) hypothetical bypass estimate (≈91 minutes earlier thrombectomy with direct routing).^12^ The relationship between delay and functional independence was parameterized using published registry estimates of reduced probability of functional independence per-hour increase from onset to EVT start (5.3% per hour).^13^ Estimated gains in functional independence per 1000 suspected stroke evaluations were derived by applying the modeled incremental number of correctly identified patients with LVO relative to the FAST-ED comparator and multiplying by the per-patient absolute benefit implied by the assumed time gain. Because not all additionally identified patients with LVO would be expected to undergo EVT and realize the full modeled time gain, an applicability factor (0.6–0.9) was applied to generate a conservative range of estimated functional independence gains.

## Results

### Patient Cohort Characteristics

In the TIME study, 382 patients with suspected stroke were recruited, and 324 comprised the final analytic cohort after prespecified exclusions.^7^ The present study reports the retrospective secondary analysis of the ≤6-hour subset (n=210), which formed the final analytic sample for LVOne validation (Figure S1). A total of 210 patients were included: 20 (9.5%) with LVO, 10 (4.8%) with ICH, 32 (15.2%) with non-LVO ischemic stroke, 8 (3.8%) with transient ischemic attack, and 140 (66.7%) with stroke mimics (including seizures, migraine, encephalopathy, hyperglycemia, hypertensive emergency, posterior reversible encephalopathy syndrome, and other undocumented causes); the detailed breakdown of mimics diagnoses is provided in Table S1. Baseline demographic and clinical characteristics are summarized in Table 1.

**Table 1.**
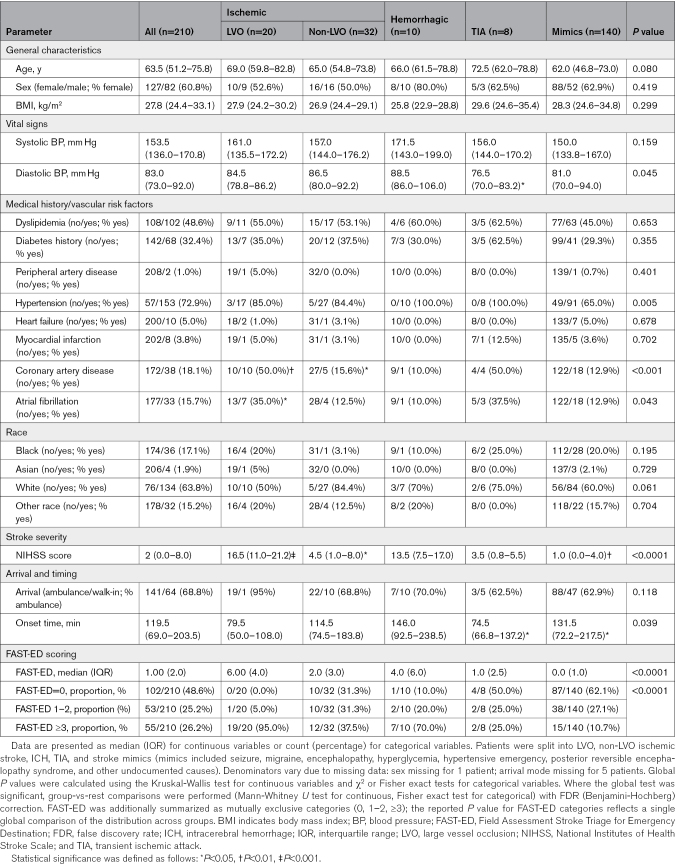
Clinical Characteristics of the Patient Cohort

Both patients with LVO and ICH presented with substantially greater stroke severity than other groups (median National Institutes of Health Stroke Scale score of 16.5 and 13.0, respectively; global *P*<0.0001). They also had shorter onset-to-arrival times (median, 79.5 and 74.5 minutes, respectively) compared with ischemic, transient ischemic attack, and mimic patients (*P*=0.039).

Risk factor profiles differed between the 2 main target groups: patients with LVO more frequently had coronary artery disease and atrial fibrillation (*q*<0.05), whereas hypertension was nearly universal in patients with ICH (*q*<0.01). Other vascular comorbidities (dyslipidemia, diabetes, peripheral artery disease, heart failure, myocardial infarction) showed no significant differences across groups.

Demographics such as age (median, 63.5 years [interquartile range, 51.2–75.8]) and sex (60.8% female overall) did not significantly differ between groups (*P*=0.08 and *P*=0.42, respectively).

### Diagnostic Performance of LVOne Versions

**Figure 3.**
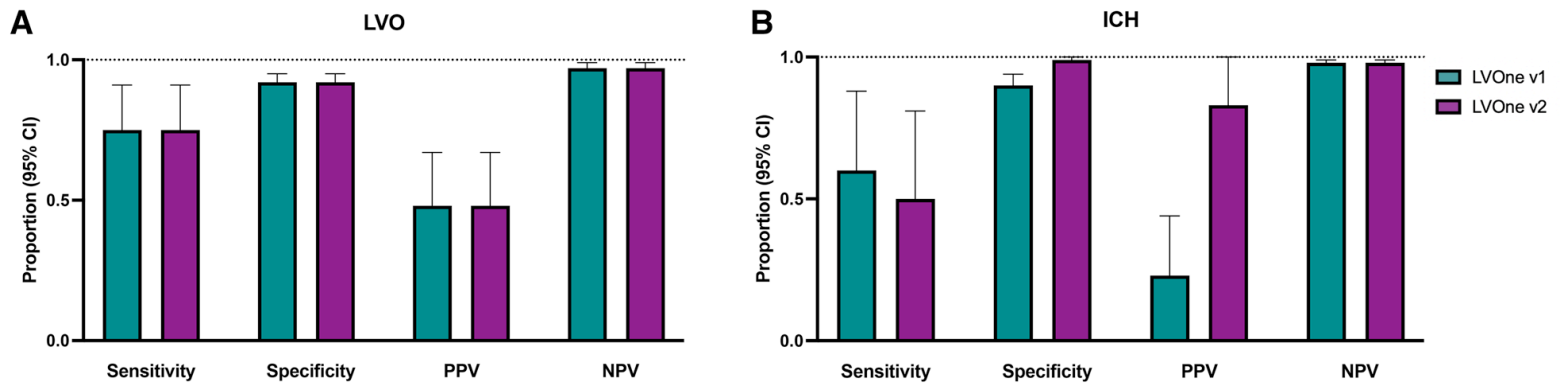
**Diagnostic performance of LVOne versions for detection of large vessel occlusion (LVO) and intracerebral hemorrhage (ICH). A**, LVO and (**B**) ICH diagnostic performance for LVOne version 1 (v1) and version 2 (v2), shown as sensitivity, specificity, positive predictive value (PPV), and negative predictive value (NPV) with 95% CIs. Bars indicate point estimates, and error bars denote 95% CIs. LVOne v2 demonstrates markedly higher specificity and PPV for ICH detection compared with v1, whereas LVO performance is similar between versions.

The diagnostic performance of the first- and second-generation LVOne tests for LVO detection was identical, replicating previously reported results in Gaude et al^10^ (Table 2). As illustrated in Figure 3, both versions demonstrated a sensitivity of 0.75 (95% CI, 0.51–0.91), specificity of 0.92 (95% CI, 0.87–0.95), PPV of 0.48 (95% CI, 0.30–0.67), and NPV of 0.97 (95% CI, 0.94–0.99).

**Table 2.**
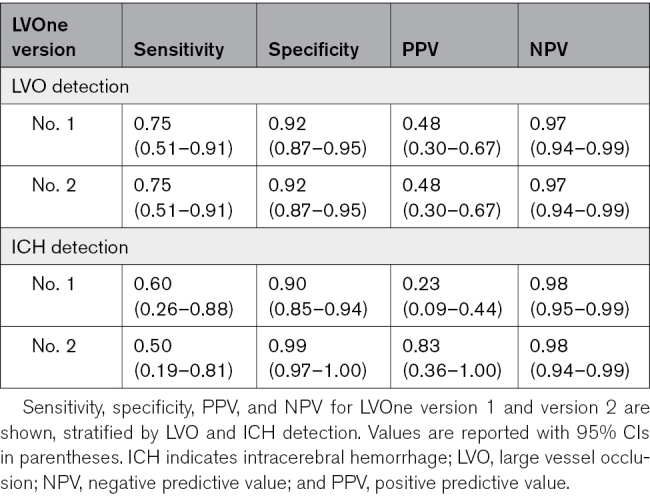
Diagnostic Performance of LVOne Versions 1 and 2 for LVO and ICH Detection

For ICH detection, the first-generation LVOne test showed a sensitivity of 0.60 (95% CI, 0.26–0.88), specificity of 0.90 (95% CI, 0.85–0.94), PPV of 0.23 (95% CI, 0.09–0.44), and NPV of 0.98 (95% CI, 0.95–0.99). The second-generation version improved specificity (0.99 [95% CI, 0.97–1.00]) and PPV (0.83 [95% CI, 0.36–1.00]), with comparable NPV (0.98 [95% CI, 0.94–0.99]), though sensitivity was slightly lower (0.50 [95% CI, 0.19–0.81]).

**Figure 4.**
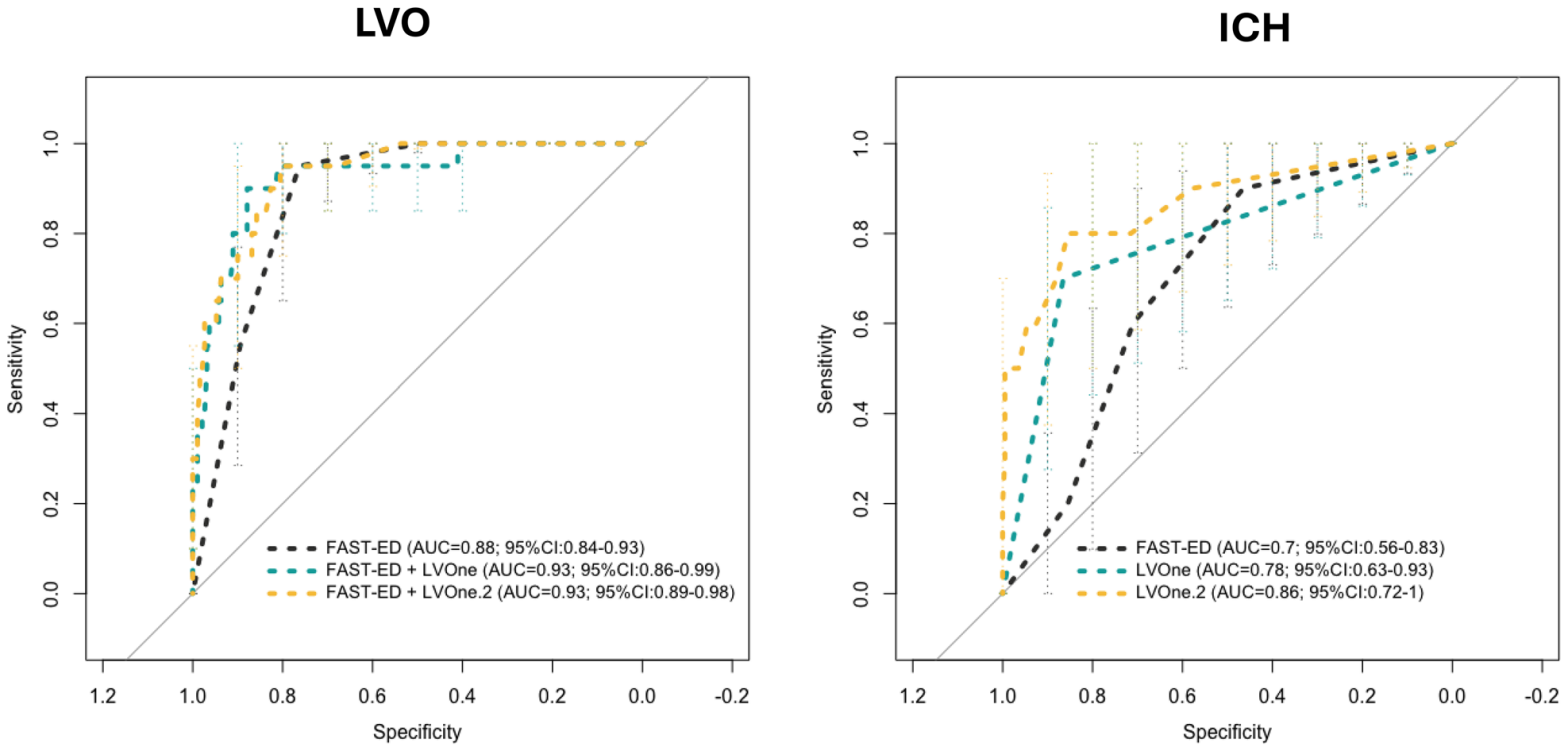
**Receiver operating characteristic (ROC) curve analyses for large vessel occlusion (LVO) and intracerebral hemorrhage (ICH) detection. Left**, ROC curves for LVO detection comparing Field Assessment Stroke Triage for Emergency Destination (FAST-ED) alone (area under the curve [AUC], 0.88 [95% CI, 0.84–0.93]) with FAST-ED plus LVOne version 1 (AUC, 0.93 [95% CI, 0.86–0.99]) and FAST-ED plus LVOne version 2 (AUC, 0.93 [95% CI, 0.89–0.98]). **Right**, ROC curve for ICH detection comparing FAST-ED alone (AUC, 0.70 [95% CI, 0.56–0.83]) with FAST-ED plus LVOne version 1 (AUC, 0.78 [95% CI, 0.63–0.93]) and FAST-ED plus LVOne version 2 (AUC, 0.86 [95% CI, 0.72–1.00]).

Receiver operating characteristic curve analysis (Figure 4) further illustrated these findings. For LVO detection, FAST-ED alone provided good discrimination (area under the curve [AUC], 0.88 [95% CI, 0.84–0.93]), which improved with the addition of LVOne biomarkers (AUC, 0.93 [95% CI, 0.86–0.99] for the first-generation and 0.93 [95% CI, 0.89–0.98] for the second-generation LVOne). The receiver operating characteristic curve for ICH detection showed that while FAST-ED alone provided limited discrimination (AUC, 0.70 [95% CI, 0.56–0.83]), the addition of LVOne improved performance (AUC, 0.78 [95% CI, 0.63–0.93]), and the second-generation LVOne assay further enhanced accuracy (AUC, 0.86 [95% CI, 0.72–1.00]), underscoring the superior discriminatory ability of LVOne v2 compared with the first-generation version.

As a sensitivity analysis, we varied the LVOne v2 d-dimer visual intensity cutoff used to define a positive result (≥1, ≥2 [benchmark], ≥3) while keeping FAST-ED ≥3 and GFAP-negative criteria fixed. Lowering the cutoff from ≥2 to ≥1 increased sensitivity from 0.75 to 0.80 but reduced specificity from 0.92 to 0.87, whereas raising the cutoff to ≥3 reduced sensitivity to 0.55 with a modest increase in specificity to 0.93; PPV remained modest (0.40–0.46), and NPV remained high (>0.95) across all thresholds (Table S4).

### Specimen-Type Comparison

In an additional experiment performed to address prehospital workflow feasibility, LVOne v2 was evaluated using venous plasma, venous whole blood, and finger-prick capillary whole blood specimens (n=25 sets). Qualitative classification (positive versus negative) for both biomarkers was concordant across all 3 specimen matrices in all sets (25/25; 100% matrix equivalence; Table S2). These data support the use of LVOne v2 with finger-prick whole blood in its intended point-of-care workflow.

### Correlation Between Biomarkers and Stroke Severity

**Figure 5.**
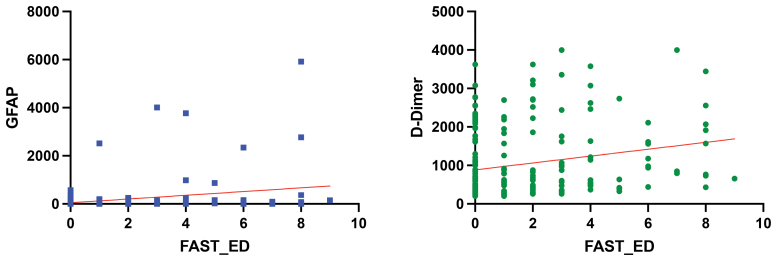
**Correlation between Field Assessment Stroke Triage for Emergency Destination (FAST-ED) scores and biomarker levels of GFAP (glial fibrillary acidic protein) and d-dimer.** Scatter plots of LVOne GFAP (**left**) and d-dimer (**right**) numerical outputs vs FAST-ED score for all participants (n=210). Red lines indicate fitted linear regression models. Both biomarkers showed statistically significant positive associations with FAST-ED (GFAP: *P*<0.0001, *R*^2^=0.077; d-dimer: *P*=0.0016, *R*^2^=0.047), but the low coefficients of determination indicate that most of the variability in biomarker values is not explained by the clinical scale, suggesting that LVOne biomarker readouts are not simply proxies for stroke severity. Equations: GFAP=77.54×FAST-ED+47.62; d-dimer=89.43×FAST-ED+883.9.

We assessed the association between stroke severity and LVOne biomarker values using linear regression models with FAST-ED score as the predictor and continuous GFAP or d-dimer signal as the outcome (Figure 5). As shown in Figure 5, both GFAP and d-dimer demonstrated statistically significant positive associations with FAST-ED scores (GFAP: *P*<0.0001, *R*^2^=0.077; d-dimer: *P*=0.0016, *R*^2^=0.047).

To further characterize the relationship between GFAP and ICH, we fitted a univariable logistic regression model with ICH as the dependent variable and log10-transformed GFAP concentration (log10[GFAP+1]) as the predictor. Higher GFAP levels were strongly associated with ICH (odds ratio per 1-unit increase in log10[GFAP+1], 10.67 [95% CI, 3.85–35.89]; *P*=0.0008), and the model showed good discrimination (AUC, 0.81 [95% CI, 0.62–1.00]; Figure S2). The predicted probability curve demonstrates a steep increase in ICH probability at higher GFAP concentrations, consistent with the role of GFAP as a marker of hemorrhagic brain injury.

### Modeled Triage Impact

Using TIME subset prevalence (LVO, 9.5%; ICH, 4.8%) and observed diagnostic performance (Table 2), LVOne v1 and v2 yielded identical modeled LVO triage outcomes (sensitivity, 0.75; specificity, 0.92), corresponding to ≈71 true-positive LVO identifications, ≈24 false negatives, and ≈72 false positives per 1000 evaluations (Table S3; Figure S3). Using published FAST-ED ≥4 performance as a scale-only comparator (sensitivity, 0.60; specificity, 0.89), FAST-ED ≥4 alone would identify ≈57 true-positive LVOs and generate ≈100 false-positive LVO activations per 1000; thus, LVOne v2 corresponds to ≈14 additional true LVO detections with ≈28 fewer false-positive bypasses relative to FAST-ED ≥4 (Table S3; Figure S3).

For ICH, LVOne v2 improved modeled operational specificity relative to v1. Under v1 performance (sensitivity, 0.60; specificity, 0.90), ≈29 of 48 expected ICH cases per 1000 would be correctly flagged with ≈95 false-positive ICH activations; in contrast, under v2 performance (sensitivity, 0.50; specificity, 0.99), ≈24 of 48 expected ICH cases would be correctly flagged with ≈10 false positives providing an absolute reduction of ≈85 unnecessary ICH-positive activations per 1000 evaluations (Table S3; Figure S3).

Applying a 91-minute time-saved scenario for direct EVT-capable routing and a 5.3% per-hour relationship between onset-to-EVT delay and functional independence, the incremental ≈14 additional LVO detections relative to FAST-ED ≥4 correspond to an estimated ≈0.7 to 1.0 additional functionally independent outcomes per 1000 suspected stroke evaluations under conservative applicability assumptions; estimates under an alternative no-bypass/frequent-transfer baseline are provided in Table S3.^12^

## Discussion

In this study, we assessed the performance of an optimized second-generation LVOne point-of-care test for the detection of LVO and ICH in patients with suspected stroke. We found that LVOne v2 significantly improved specificity and PPV for ICH detection, while maintaining the high sensitivity and specificity for LVO observed with the first-generation test.^10^ Importantly, when used in combination with established clinical assessment tools such as the FAST-ED score, both LVOne versions achieved high NPV across both stroke subtypes, supporting potential utility as adjunctive triage tools (noting that PPV/NPV are prevalence-dependent). These findings continue to suggest that LVOne could ultimately be suited for prehospital application, where early identification or exclusion of LVO and ICH may substantially influence acute stroke management strategies and optimize triage decisions regarding hospital destination. However, we note that the present validation was conducted in patient plasma; further studies are required to confirm performance in capillary whole blood and in real-world prehospital environments before clinical deployment. Current prehospital triage algorithms rely heavily on clinical scales, which have limited diagnostic accuracy and provide only indirect, nonspecific information about stroke subtype. To date, few blood-based point-of-care approaches have demonstrated clinically actionable dual-subtype (LVO versus ICH) discrimination in a workflow intended for prehospital triage; therefore, LVOne represents a leading candidate for prospective field validation.

### Clinical and System Implications

The ability to accurately distinguish LVO from ICH in the prehospital setting carries critical clinical and logistical implications. Timely identification of LVO is essential, as these patients require emergent transfer to comprehensive stroke centers for mechanical thrombectomy, a time-sensitive intervention that significantly improves outcomes.^14^ In contrast, most patients with ICH are best managed at primary stroke centers, where early medical stabilization, particularly, intensive blood pressure control, can mitigate hematoma expansion and improve recovery, as demonstrated in the INTERACT4 trial (Intensive Ambulance-Delivered Blood Pressure Reduction in Hyper-Acute Stroke).^4^ Importantly, early prehospital identification of ICH may facilitate blood pressure management even before hospital arrival, a strategy gaining increased attention for integration into acute stroke protocols.^15^ Inappropriate triage of patients with ICH to distant comprehensive stroke centers can be harmful, as suggested by the RACECAT trial (Randomized Acute Cerebral Embolism Clinical Approach Trial), where longer transfer times delayed initiation of critical therapies and were associated with worse outcomes in non-LVO stroke populations.^16^

Beyond patient-level benefits, precise triage has substantial system-level implications. Studies have shown that patients with LVO initially transported to primary stroke centers and later transferred for thrombectomy experience delayed treatment and reduced odds of functional independence at 90 days.^17^ Inappropriate triaging also places a significant financial burden on healthcare systems. Depending on patient selection and recanalization probability, the incremental cost of interhospital transfer for thrombectomy can reach up to $20 000 per patient, with cost-effectiveness ratios rising as high as $310 000 per quality-adjusted life years gained in lower-benefit scenarios.^18^ By ensuring that the right patient reaches the right facility on the first attempt, biomarker-based diagnostic tools have the potential to improve both patient outcomes and system efficiency.

### Modeling Clinical Impact

In modeling based on the TIME ≤6-hour prevalence and observed diagnostic accuracy, LVOne v2 modestly increased correct LVO identification while reducing unnecessary bypass activations compared with a scale-only comparator (FAST-ED≥4) and substantially reduced false-positive ICH-positive activations compared with LVOne v1 (Table S3; Figure S3). Operationally, improved LVO identification is most relevant in systems where correct field recognition can trigger direct routing to EVT-capable centers and reduce secondary-transfer delays, whereas the higher specificity for ICH is particularly important for limiting inappropriate diversion/activation and preserving stroke-system capacity while maintaining high NPV. Early identification of likely ICH may also support earlier initiation of hemorrhage-focused pathways, including timely blood pressure management, as evaluated in early treatment paradigms such as INTERACT4.^4^ The time-outcome translation is intended to be illustrative: expected clinical benefit depends on regional prevalence, bypass thresholds, geography, and the proportion of additionally identified patients with LVO who are EVT-eligible and who realize a meaningful time gain. Accordingly, these modeling outputs should be interpreted as scenario estimates that motivate prospective field validation and health-system evaluation rather than definitive outcome predictions.

### Biological Rationale and Implementation Considerations

Beyond the specific improvements achieved in LVOne v2, the broader concept underlying the platform warrants emphasis. Prior studies, including the TIME study and the initial validation by Gaude et al,^10^ have shown that combining plasma d-dimer and GFAP measurements enables clinically meaningful differentiation between LVO and ICH. GFAP rises rapidly in ICH due to acute astrocytic injury but remains low in LVO during the early triage window.^9,19^ Meta-analyses show GFAP distinguishes ICH from ischemic stroke with high sensitivity (≈78%) and specificity (≈95%).^20^ Conversely, d-dimer is typically elevated in LVO versus other suspected stroke subtypes.^8,21^ Therefore, biomarker-based approaches address a critical limitation of current prehospital stroke triage, where reliance on clinical exam-based scales—such as FAST-ED, Rapid Arterial Occlusion Evaluation, and Los Angeles Motor Scale, which are among the most widely used tools in emergency medical services settings—yields only moderate diagnostic accuracy, particularly in distinguishing stroke subtypes.^6,22^

Importantly, the LVOne test has several practical advantages that support its potential scalability and real-world utility. The assay is rapid, requires only a small blood sample, and does not rely on specialized laboratory infrastructure or advanced operator training. Hence, we agree with other groups that these features make it particularly well-suited for point-of-care deployment in prehospital settings, aligning with the pressing need for portable, reliable, and easy-to-use diagnostic tools in acute stroke care.^23,24^ Taken together, the accumulated evidence supports the promise of the LVOne platform as an adjunctive diagnostic tool to improve stroke triage, reduce misdiagnosis, and ultimately enhance patient outcomes on a system-wide scale. Specifically, minimal infrastructure requirements may offer unique value in low-resource or rural settings, where timely neuroimaging is unavailable, and stroke care disparities remain pronounced.

### Limitations

The present study has several limitations. Most notably, the LVO and ICH subgroup sample size was small, and the retrospective design introduces potential selection and information biases. However, this also reflects the real-world prevalence of LVO in unselected stroke alert populations (≈10%), and the absolute sample size is consistent with similar diagnostic studies.^6,7^ Although biomarker assessments were performed blinded, the nature of d-dimer scoring may allow for some operator bias. In addition, although the TIME cohort simulated prehospital conditions, real-world operational feasibility was not assessed, and the single-system setting may limit generalizability. In addition, meta-analyses validating GFAP as an ICH biomarker have noted its time-dependent characteristics. In LVO, GFAP levels remain low early on but can approach ICH-like levels several hours after symptom onset.^25^ Therefore, if stroke recognition is delayed, the diagnostic specificity of GFAP may diminish, increasing the risk of misclassification. Moreover, although scoring was blinded and performed using a reference standard, the nature of d-dimer interpretation introduces potential for inter-operator variability, particularly in borderline results. Prospective validation in diverse healthcare environments is warranted.

### Strengths and Future Directions

This study leverages the well-characterized TIME cohort, with plasma collected before thrombolysis or imaging, effectively simulating real-world prehospital stroke populations. Direct comparison of 2 LVOne versions allowed evaluation of both reproducibility and diagnostic improvements, while dual GFAP and d-dimer measurement enabled assessment of combined biomarker performance for LVO and ICH detection.

Future studies should prospectively validate LVOne in prehospital and emergency settings, assess usability by frontline providers, and evaluate generalizability through multicenter trials. Health-economic analyses, regulatory planning, and integration with digital triage tools will be key to supporting scalable deployment.

### Conclusions

The optimized v2 of the LVOne test retains the LVO detection performance of the original assay while significantly enhancing the detection of hemorrhagic strokes. By enabling reliable prehospital differentiation of both LVO and ICH, LVOne represents the first blood-based point-of-care platform with validated dual-subtype diagnostic potential. These findings suggest that LVOne could meaningfully improve stroke care by supporting earlier and more accurate diagnosis, guiding triage and transfer decisions, and ultimately facilitating timely, tailored treatment interventions in the acute setting.

## ARTICLE INFORMATION

### Disclosures

Dr Bernstock has an equity position in Treovir, Inc, and is Chief Medical Officer of UpFront Diagnostics and Centile Bioscience. Dr Bernstock is also on the QV Bioelectronics and NeuroX1 boards of scientific advisors and the Synaptive Medical Board. Dr Gaude is Chief Scientific Officer of Upfront Diagnostics. The other authors report no conflicts.

### Supplemental Material

Supplemental Methods

Tables S1–S4

Figures S1–S3

STROBE Checklist

## Supplementary Material
