# Supplementary material for: Prehospital Detection of Large Vessel Occlusion and Intracerebral Hemorrhage Using a Dual-Biomarker Point-of-Care Test
Source: Stroke Vasc Interv Neurol. 2026 Mar 2;6(3):e002170. doi: 10.1161/SVIN.125.002170 (PMC13138425; doi:10.1161/SVIN.125.002170)
Supplement: Supplementary file 1 [file svi2-6-e002170-s001.pdf]

SUPPLEMENTAL MATERIALS

Prehospital Detection of Large Vessel Occlusion and Intracerebral Hemorrhage Using a Dual-Biomarker Point-of-Care Test.

Authors: Arnab Ghosh, BSc[1*]; Noah L.A. Nawabi, BS[2,3*]; Diana Alcedo, MS[4]; Rodolfo E. Alcedo Guardia, MD[5]; Juan Vicenty-Padilla, MD[5]; Saef Izzy, MD[6]; Nirav J. Patel, MD[7]; Rose Du, MD, PhD[7]; Adam A. Dmytriw, MD[8]; Alfred P. See, MD[9]; Mohammed Ali Aziz-Sultan, MD, MBA[7]; Toby I. Gropen, MD[10]; David Liebeskind, MD[11]; Erickson F. Torio, MD[7]; Anil Can, MD[7]; Lennard Spanehl, MD[7]; Edoardo Gaude, PhD[12**]; Joshua D. Bernstock, MD, PhD, MPH[7**]

*AG and NLAN are co-first authors

**EG and JDB are co-senior authors

Correspondence: Edoardo Gaude; Joshua D. Bernstock

Affiliations:

1 Medical Sciences Division, University of Oxford, Oxford, UK

2 Computational Neuroscience Outcomes Center, Department of Neurosurgery, Brigham and Women’s Hospital, Harvard Medical School, Boston, MA, USA

3 College of Medicine, Medical University of South Carolina, Charleston, SC

4 Ponce Health Sciences University, Ponce, Puerto Rico

5 Neurosurgery Section, School of Medicine University of Puerto Rico, Medical Sciences Campus, San Juan, PR

6 Department of Neurology, Brigham and Women’s Hospital, Boston, MA

7 Department of Neurosurgery, Brigham and Women’s Hospital, Boston, MA

8 Neuroendovascular Program, Massachusetts General Hospital, Harvard Medical School, Boston MA, USA.

9 Department of Neurosurgery, Boston Children’s Hospital, Boston, MA

10 Department of Neurology, The University of Alabama at Birmingham Heersink School of Medicine, Birmingham, AL

11 Comprehensive Stroke Center and Department of Neurology, University of California, Los Angeles, California, United States of America.

12 Pockit diagnostics Ltd, University of Cambridge, CRUK Cambridge Institute, Cambridge, UK

Major Resources Table

Resource Type	Resource / Description	Source / Provider	Identifier / URL	Notes
Human cohort	TIME suspected stroke cohort (Brandon Regional Hospital)	Durrani et al. 2024; Brandon Regional Hospital, FL	ClinicalTrials.gov NCT04292600	Prospective observational cohort of suspected stroke patients enrolled within 18 hours of symptom onset.
Biospecimens	Archived venous plasma samples from TIME biorepository	TIME biorepository, Brandon Regional Hospital	N/A	Plasma obtained prior to thrombolysis or neuroimaging and stored at −80 °C until analysis.
Diagnostic assay	LVOne Version 1 (GFAP + D-dimer point-of-care test)	Pockit Diagnostics Ltd, Cambridge, UK	Catalogue PDL-002, LVOne v1	First-generation dual-biomarker lateral-flow assay; D-dimer positivity defined per manufacturer threshold.
Diagnostic assay	LVOne Version 2 (GFAP + D-dimer point-of-care test)	Pockit Diagnostics Ltd, Cambridge, UK	Catalogue PDL-002, LVOne v2	Second-generation LVOne with updated GFAP/D-dimer thresholds; evaluated in this study.
Clinical scale	FAST-ED (Field Assessment Stroke Triage for Emergency Destination)	Lima et al. 2016	N/A	Prehospital stroke severity scale used to define FAST-ED ≥3 (LVO) and ≥1 (ICH) thresholds in decision rule.
Imaging modality	CTA and MRA for LVO confirmation; non-contrast CT/MRI for ICH	Standard-of-care hospital scanners	N/A	Vascular and parenchymal imaging used as reference standard for final diagnostic category.
Statistical software	R (statistical computing environment)	R Foundation for Statistical Computing	Version 4.4.1	Used for all statistical analyses (diagnostic performance, logistic regression, triage modelling).

Supplemental Table S1: Data for Participants with Expanded Mimics.

Parameter	All (n=210)	Ischemic		Hemorrhagic (n=10)	TIA (n=8)	Mimics				P-value
		LVO (n=20)	Non-LVO (n=32)			Seizure (n=11)	Migraine (n=10)	Encephalopathy (n=18)	Others (n=101)	
General Characteristics										
Age, years	63.5 (51.2–75.8)	69.0 (59.8–82.8)	65.0 (54.8–73.8)	66.0 (61.5–78.8)	72.5 (62.0-78.8)	68.0 (49.5–75.5)	44.0 (43.2–45.0)	71.0 (62.2–84.0)	62.0 (48.0–73.0)	0.25
Sex (female/male; % female)	127/82 (60.8%)	10/9 (52.6%)	16/16 (50.0%)	8/10 (80.0%)	5/3 (62.5%)	8/3 (72.7%)	9/1 (90.0%)	14/4 (77.8%)	44/57 (56.4%)	0.91
BMI, kg/m²	27.8 (24.4–33.1)	27.9 (24.2–30.2)	26.9 (24.4–29.1)	25.8 (22.9–28.8)	29.6 (24.6-35.4)	28.3 (24.5–33.5)	30.2 (28.4–46.6)	23.8 (20.9–25.8)	28.5 (25.1–35.4)	0.90
Vital Signs										
Systolic BP, mmHg	153.5 (136.0–170.8)	161.0 (135.5–172.2)	157.0 (144.0–176.2)	171.5 (143.0–199.0)	156.0 (144.0–170.2)	138.0 (131.0–150.0)	151.5 (143.0–160.8)	145.0 (123.2–165.5)	154.0 (135.0–170.0)	0.90
Diastolic BP, mmHg	83.0 (73.0–92.0)	84.5 (78.8–86.2)	86.5 (80.0–92.2)	88.5 (86.0–106.0)	76.5 (70.0–83.2)	82.0 (71.0–88.5)	90.5 (73.0–94.8)	68.5 (62.5–87.8)	81.0 (73.0–94.0)	0.98
Medical History / Vascular Risk Factors										
Dyslipidemia (no/yes; % yes)	108/102 (48.6%)	9/11 (55.0%)	15/17 (53.1%)	4/6 (60.0%)	3/5 (62.5%)	5/6 (54.5%)	8/2 (20.0%)	8/10 (55.6%)	56/45 (44.6%)	0.98
Diabetes history (no/yes; % yes)	142/68 (32.4%)	13/7 (35.0%)	20/12 (37.5%)	7/3 (30.0%)	3/5 (62.5%)	7/4 (36.4%)	9/1 (10.0%)	12/6 (33.3%)	71/30 (29.7%)	1.00
Peripheral arterial disease (no/yes; % yes)	208/2 (1.0%)	19/1 (5.0%)	32/0 (0.0%)	10/0 (0.0%)	8/0 (0.0%)	11/0 (0.0%)	10/0 (0.0%)	18/0 (0.0%)	100/1 (1.0%)	0.90

Hypertension (no/yes; % yes)	57/153 (72.9%)	3/17 (85.0%)	5/27 (84.4%)	0/10 (100.0%)	0/8 (100.0%)	4/7 (63.6%)	7/3 (30.0%)	4/14 (77.8%)	34/67 (66.3%)	0.81
Heart failure (no/yes; % yes)	200/10 (5.0%)	18/2 (1.0%)	31/1 (3.1%)	10/0 (0.0%)	8/0 (0.0%)	11/0 (0.0%)	10/0 (0.0%)	16/2 (11.1%)	96/5 (5.0%)	0.90
Myocardial infarction (no/yes; % yes)	202/8 (3.8%)	19/1 (5.0%)	31/1 (3.1%)	10/0 (0.0%)	7/1 (12.5%)	10/1 (9.1%)	10/0 (0.0%)	18/0 (0.0%)	97/4 (4.0%)	1.00
Coronary artery disease (no/yes; % yes)	172/38 (18.1%)	10/10 (50.0%)	27/5 (15.6%)	9/1 (10.0%)	4/4 (50.0%)	7/4 (36.4%)	9/1 (10.0%)	17/1 (5.6%)	89/12 (11.9%)	0.0035 **
Atrial fibrillation (no/yes; % yes)	177/33 (15.7%)	13/7 (35.0%)	28/4 (12.5%)	9/1 (10.0%)	5/3 (37.5%)	9/2 (18.2%)	10/0 (0.0%)	14/4 (22.2%)	89/12 (11.9%)	0.13
Ethnicity										
African American (no/yes; % yes)	174/36 (17.1%)	16/4 (20%)	31/1 (3.1%)	9/1 (10.0%)	6/2 (25.0%)	7/4 (36.4%)	10/0 (0.0%)	17/1 (5.6%)	78/23 (22.8%)	1.00
Asian (no/yes; % yes)	206/4 (1.9%)	19/1 (5%)	32/0 (0.0%)	10/0 (0.0%)	8/0 (0.0%)	11/0 (0.0%)	10/0 (0.0%)	18/0 (0.0%)	98/3 (3.0%)	0.98
Caucasian (no/yes; % yes)	76/134 (63.8%)	10/10 (50%)	5/27 (84.4%)	3/7 (70%)	2/6 (75.0%)	4/7 (63.6%)	4/6 (60.0%)	5/13 (72.2%)	43/58 (57.4%)	0.81
Other ethnicity (no/yes; % yes)	178/32 (15.2%)	16/4 (20%)	28/4 (12.5%)	8/2 (20%)	8/0 (0.0%)	11/0 (0.0%)	6/4 (40.0%)	15/3 (16.7%)	86/15 (14.9%)	0.98
Stroke Severity										
NIHSS score	2 (0.0–8.0)	16.5 (11.0–21.2)	4.5 (1.0–8.0)	13.5 (7.5–17.0)	3.5 (0.8-5.5)	2.0 (0.5–5.0)	2.0 (1.2–3.8)	2.5 (0.0–9.0)	1.0 (0.0–3.0)	<0.000001 ***
Arrival and Timing										
Arrival (ambulance/walk-in; % ambulance)	141/64 (68.8%)	19/1 (95%)	22/10 (68.8%)	7/10 (70.0%)	3/5 (62.5%)	2/9 (81.8%)	5/5 (50.0%)	4/14 (77.8%)	60/37 (61.9%)	0.08

Onset time, min	119.5 (69.0–203.5)	79.5 (50.0–108.0)	114.5 (74.5–183.8)	146.0 (92.5–238.5)	74.5 (66.8-137.2)	180.0 (75.0–269.2)	171.5 (98.2–218.0)	85.0 (53.2–206.5)	123 (73.0–214.0)	0.04 *
FAST-ED Scoring										
FAST-ED, median (IQR)	1.00 (2.0)	6.00 (4.0)	2.0 (3.0)	4.0 (6.0)	1.0 (2.5)	0.0 (1.0)	0.5 (1.0)	0.5 (3.0)	0.0 (1.0)	<0.0001 ****
FAST-ED = 0, proportion (%)	102/210 (48.6%)	0/20 (0.0%)	10/32 (31.3%)	1/10 (10.0%)	4/8 (50.0%)	8/11 (72.7%)	5/10 (50.0%)	9/18 (50.0%)	65/101 (64.4%)	<0.0001 ****
FAST-ED 1-2, proportion (%)	53/210 (25.2%)	1/20 (5.0%)	10/32 (31.3%)	2/10 (20.0%)	2/8 (25.0%)	1/11 (9.1%)	5/10 (50.0%)	4/18 (22.2%)	28/101 (27.7%)	
FAST-ED ≥ 3, proportion (%)	55/210 (26.2%)	19/20 (95.0%)	12/32 (37.5%)	7/10 (70.0%)	2/8 (25.0%)	2/11 (18.2%)	0/10 (0.0%)	5/18 (27.8%)	8/101 (7.9%)	

Baseline clinical and imaging characteristics for the LVOne v2 ≤6-hour cohort (n=210), stratified by final diagnostic category (ischemic stroke, intracerebral hemorrhage, transient ischemic attack, and stroke mimics). Values are reported as median (interquartile range) or n (%). P values correspond to global comparisons across diagnostic groups using the Kruskal–Wallis test for continuous variables and Fisher's exact test for categorical variables. LVO indicates large vessel occlusion; ICH, intracerebral hemorrhage; TIA, transient ischemic attack.

Supplemental Table S2. Specimen-type comparison of LVOne v2 readouts across venous plasma, venous whole blood, and finger-prick whole blood.

Sample	D-dimer						GFAP					
	Fingerprick Blank	Fingerprick Spiked	Venous whole blood Blank	Venous whole blood Spiked	Venous plasma Blank	Venous plasma Spiked	Fingerprick Blank	Fingerprick Spiked	Venous whole blood Blank	Venous whole blood Spiked	Venous plasma Blank	Venous plasma Spiked
1	*1*	*8*	*0*	*5*	*0*	*8*	*Negative*	*Positive*	*Negative*	*Positive*	*Negative*	*Positive*
2	*0*	*7*	*0*	*8*	*0*	*8*	*Negative*	*Positive*	*Negative*	*Positive*	*Negative*	*Positive*
3	*0*	*5*	*0*	*8*	*0*	*8*	*Negative*	*Positive*	*Negative*	*Positive*	*Negative*	*Positive*
4	*0*	*5*	*0*	*8*	*0*	*8*	*Negative*	*Positive*	*Negative*	*Positive*	*Negative*	*Positive*
5	*0*	*8*	*1*	*7*	*0*	*7*	*Negative*	*Positive*	*Negative*	*Positive*	*Negative*	*Positive*
6	*0*	*8*	*0*	*8*	*0*	*8*	*Negative*	*Positive*	*Negative*	*Positive*	*Negative*	*Positive*
7	*0*	*4*	*0*	*8*	*0*	*4*	*Negative*	*Positive*	*Negative*	*Positive*	*Negative*	*Positive*
8	*0*	*4*	*1*	*7*	*0*	*4*	*Negative*	*Positive*	*Negative*	*Positive*	*Negative*	*Positive*
9	*0*	*5*	*0*	*8*	*0*	*5*	*Negative*	*Positive*	*Negative*	*Positive*	*Negative*	*Positive*
10	*1*	*5*	*1*	*6*	*1*	*6*	*Negative*	*Positive*	*Negative*	*Positive*	*Negative*	*Positive*
11	*0*	*8*	*0*	*7*	*0*	*8*	*Negative*	*Positive*	*Negative*	*Positive*	*Negative*	*Positive*
12	*0*	*4*	*0*	*8*	*0*	*4*	*Negative*	*Positive*	*Negative*	*Positive*	*Negative*	*Positive*
13	*0*	*4*	*0*	*8*	*0*	*4*	*Negative*	*Positive*	*Negative*	*Positive*	*Negative*	*Positive*
14	*0*	*2*	*0*	*8*	*0*	*2*	*Negative*	*Positive*	*Negative*	*Positive*	*Negative*	*Positive*
15	*0*	*3*	*0*	*8*	*0*	*3*	*Negative*	*Positive*	*Negative*	*Positive*	*Negative*	*Positive*
16	*0*	*4*	*0*	*8*	*0*	*4*	*Negative*	*Positive*	*Negative*	*Positive*	*Negative*	*Positive*
17	*0*	*4*	*0*	*8*	*0*	*4*	*Negative*	*Positive*	*Negative*	*Positive*	*Negative*	*Positive*
18	*0*	*4*	*0*	*8*	*0*	*4*	*Negative*	*Positive*	*Negative*	*Positive*	*Negative*	*Positive*
19	*0*	*4*	*0*	*7*	*0*	*4*	*Negative*	*Positive*	*Negative*	*Positive*	*Negative*	*Positive*
20	*0*	*4*	*0*	*8*	*1*	*6*	*Negative*	*Positive*	*Negative*	*Positive*	*Negative*	*Positive*
21	*0*	*4*	*0*	*4*	*0*	*6*	*Negative*	*Positive*	*Negative*	*Positive*	*Negative*	*Positive*
22	*0*	*8*	*0*	*5*	*0*	*5*	*Negative*	*Positive*	*Negative*	*Positive*	*Negative*	*Positive*
23	*0*	*8*	*1*	*5*	*0*	*5*	*Negative*	*Positive*	*Negative*	*Positive*	*Negative*	*Positive*
24	*0*	*4*	*0*	*8*	*0*	*8*	*Negative*	*Positive*	*Negative*	*Positive*	*Negative*	*Positive*
25	*0*	*5*	*0*	*4*	*0*	*4*	*Negative*	*Positive*	*Negative*	*Positive*	*Negative*	*Positive*

Matched specimens were tested using LVOne v2. Values indicate the number of replicate reads classified as Negative or Positive for each biomarker and specimen type. This comparison was performed to assess feasibility of intended point-of-care use with

finger-prick whole blood; the primary diagnostic validation in the main manuscript was performed using archived venous plasma from the TIME biorepository.

Supplemental Table S3. Modeled triage impact of LVOne v1 vs v2 (and FAST-ED comparator) per 1,000 suspected stroke patients (base case uses TIME subset prevalence)

Target condition	Triage strategy	Prevalence assumed	Sensitivity	Specificity	TP (correctly flagged)	FP (unnecessary activation/diversion)	FN (missed)	TN (correctly not flagged)	Total "positive triage" (TP+FP)
LVO	FAST-ED ≥4 (scale-only comparator)	9.5%	0.60	0.89	57	100	38	805	157
	LVOne v1 decision rule		0.75	0.92	71	72	24	833	143
	LVOne v2 decision rule		0.75	0.92	71	72	24	833	143
ICH	LVOne v1 decision rule	4.8%	0.60	0.90	29	95	19	857	124
	LVOne v2 decision rule		0.50	0.99	24	10	24	942	34

Data are modeled per 1,000 suspected stroke evaluations using base-case prevalences from the TIME subset (LVO 20/210 = 9.5%; ICH 10/210 = 4.8%) and point-estimate sensitivities/specificities from the present validation. "Positive triage" represents the number of patients who would trigger the relevant destination/pathway/activation under each strategy (e.g., EVT-capable pathway for LVO; hemorrhage pathway for ICH), which may vary by local EMS protocol. Counts were calculated as: TP = N × Prev × Se; FN = N × Prev × (1–Se); FP = N × (1–Prev) × (1–Sp); TN = N – TP – FN – FP (rounded to the nearest whole number before deriving TN to ensure totals sum to 1,000). The FAST-ED ≥4 row is included as a context comparator based on commonly cited performance in published FAST-ED validation studies; performance varies by setting and implementation. For time–outcome translation, an applicability factor of ***0.6–0.9*** *was used to reflect that not all additionally identified LVO patients would (i) undergo EVT and (ii) realize the full modeled time gain; estimates therefore represent illustrative ranges. These estimates are illustrative and do not incorporate transport-time thresholds, IVT/EVT eligibility, reperfusion probability, or regional workflow constraints.*

Supplemental Table S4. Sensitivity analysis of LVOne v2 LVO triage performance across alternative D-dimer intensity thresholds.

D-dimer Positivity	*LVOne v2 (FAST-ED≥3)*			
	Sensitivity	***Specificity***	***PPV***	***NPV***
≥1 (v2)	*0.80 (0.58–0.92)*	*0.87 (0.82–0.91)*	*0.40 (0.26–0.55)*	*0.98 (0.94–0.99)*
≥2 (v2) Benchmark	*0.75 (0.53–0.89)*	*0.92 (0.87–0.95)*	*0.50 (0.33–0.67)*	*0.97 (0.94–0.99)*
≥3 (v2)	*0.55 (0.34–0.74)*	*0.93 (0.89–0.96)*	*0.46 (0.28–0.65)*	*0.95 (0.91–0.97)*

Performance of the LVOne v2 rule for detection of LVO in the ≤6-hour cohort when varying the D-dimer visual intensity cut-off, while keeping FAST-ED (≥3) and GFAP (negative) criteria unchanged. LVOne v1 sensitivity across corresponding thresholds is reported in Gaude et al. 2025 for comparison. Values are shown as proportion (95% confidence interval), with 95% CIs calculated using Wilson's method. FAST-ED indicates Field Assessment Stroke Triage for Emergency Destination; GFAP, glial fibrillary acidic protein; LVO, large vessel occlusion; NPV, negative predictive value; PPV, positive predictive value.

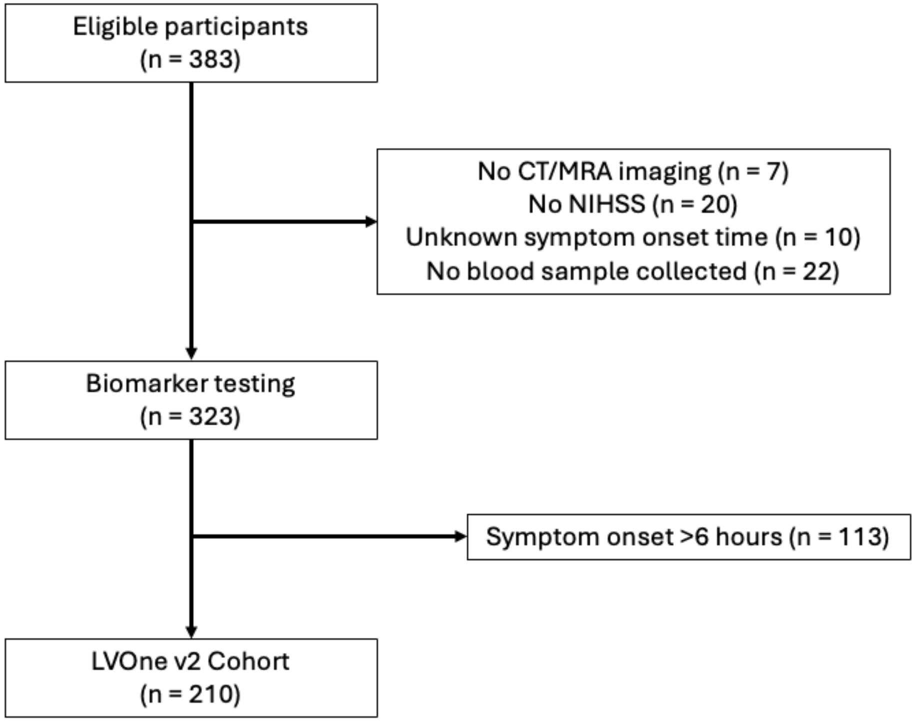

Supplemental Figure S1. Participant flow diagram for derivation of the LVOne v2 analytic cohort.

Flow diagram showing derivation of the LVOne v2 analytic cohort from the parent TIME study population. Eligible participants were excluded for missing neuroimaging (CT/CT angiography or MR angiography), missing NIHSS, unknown symptom onset time, or no blood sample collected, yielding the biomarker-testing cohort (n = 324). Participants presenting >6 hours from symptom onset were then excluded (n = 114), resulting in the final LVOne v2 cohort (n = 210). Adapted from Durrani et al., 2024. Abbreviations: CT, computed tomography; MRA, magnetic resonance angiography; NIHSS, National Institutes of Health Stroke Scale.

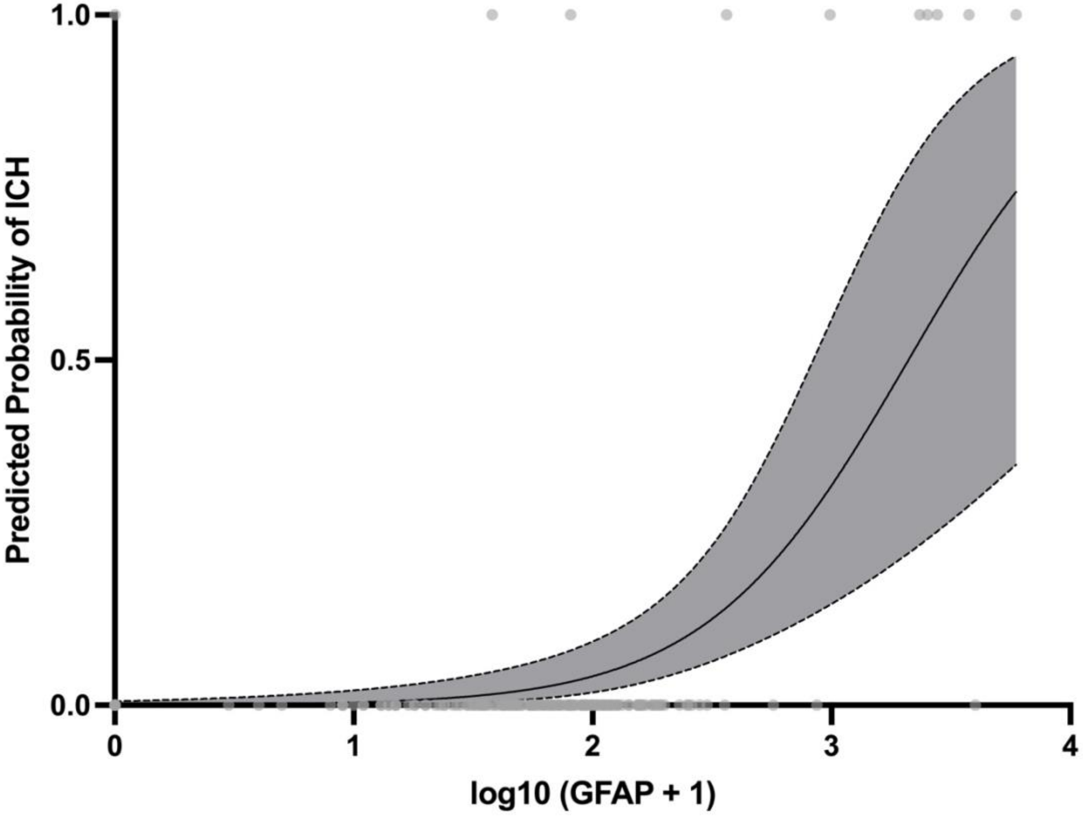

Supplemental Figure S2. Logistic regression of intracerebral hemorrhage (ICH) on LVOne GFAP concentration.

Predicted probability of ICH as a function of log10-transformed LVOne GFAP concentration (log10[GFAP + 1]) from a univariable logistic regression model. The solid line represents the fitted probability of ICH; the shaded grey band shows the 95% confidence interval; grey points represent individual observations. Each 1-unit increase in log10(GFAP + 1) was associated with higher odds of ICH (odds ratio 10.67; 95% CI 3.85–35.89; p = 0.0008), and the model showed good discrimination (AUC 0.81; 95% CI 0.62–1.00).

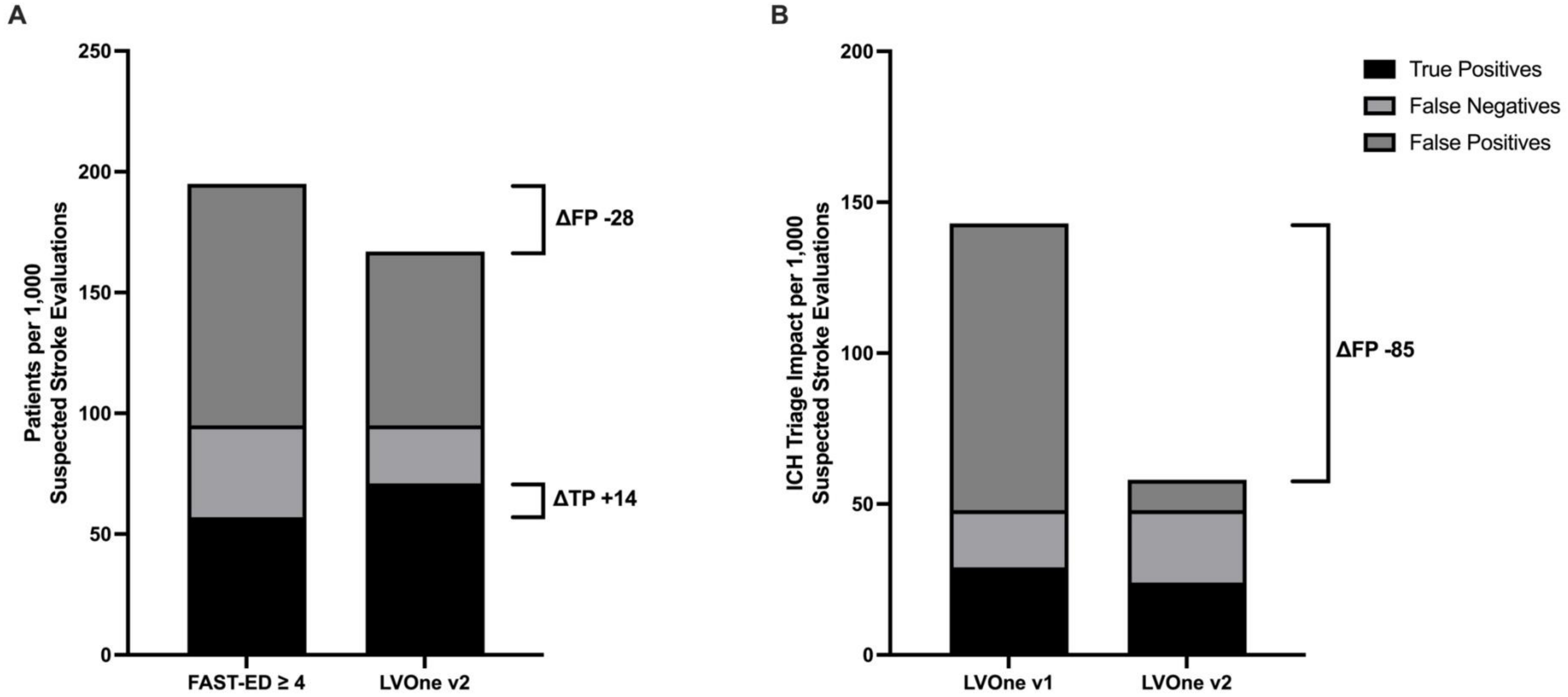

Supplemental Figure S3. Modelled triage impact of LVOne-based pathways in suspected stroke (per 1,000 evaluations).

Stacked bar charts show the expected number of true positives (TP), false negatives (FN), and false positives (FP) per 1,000 suspected stroke evaluations, calculated using the observed subtype prevalence in the TIME ≤6-hour subset (LVO 9.5%, ICH 4.8%) and the diagnostic performance estimates reported in Table 2. ***(A) LVO triage****: comparison of a scale-only strategy (FAST-ED ≥4) versus the LVOne v2 decision rule.* ***(B) ICH triage****: comparison of LVOne v1 versus LVOne v2. Model inputs, formulas, rounding conventions, and time–outcome translation assumptions are detailed in* ***Supplemental Table S3****.*
